# Eosinophilic gastroenteritis in a 14-year-old patient with Noonan syndrome with a *PTPN11* variation: a case report

**DOI:** 10.1186/s13256-025-05344-6

**Published:** 2025-06-13

**Authors:** Nobuhiko Koga, Shuichi Yatsuga, Kei Kubota, Toshikazu Niimi, Takahito Inoue, Shinichiro Nagamitsu

**Affiliations:** Department of Pediatrics, Fukuoka University School of Medicine, 7–45–1, Nanakuma, Jonan-Ku, Fukuoka City, Fukuoka 814-0180 Japan

**Keywords:** Noonan syndrome, Eosinophilic gastroenteritis, *PTPN11* gene, Allergy

## Abstract

**Background:**

Noonan syndrome has a wide range of symptoms due to dysregulation of the RAS/MAPK pathway with several gene variations, including the *PTPN11* gene. There are currently no case reports of Noonan syndrome with eosinophilic gastroenteritis.

**Case:**

A 14-year-old Japanese girl was clinically diagnosed with Noonan syndrome. She had intermittent abdominal pain and vomiting from 10 years old. The patient was diagnosed with eosinophilic gastroenteritis on the basis of the pathological finding of multiple foci with > 20 eosinophils/high power field in the mucosal lamina propria of the colon by endoscopy at 12 years old. Vomiting and abdominal pain are currently being controlled by antihistamines and leukotriene antagonist therapy. Genetic testing showed the missense variation *p.Ala72Gly* in the *PTPN11* gene.

**Discussion:**

The pathogenesis of eosinophilic gastroenteritis is similar to that of other allergic inflammatory diseases, such as bronchial asthma. The cause of eosinophilic gastroenteritis is multifactorial, including genetic and environmental factors. The *PTPN11* gene variations are suggested to promote eosinophilic disorders by leading to the activation of the RAS/MAPK pathway. This activation subsequently results in the production of interleukin-5, which plays a crucial role in the pathogenesis of eosinophilic gastroenteritis. The relationship between eosinophilic gastroenteritis and the *PTPN11* gene has not yet been reported.

**Conclusion:**

We herein present the first known case of eosinophilic gastroenteritis in Noonan syndrome with a variation in the *PTPN11* gene. The relationship between Noonan syndrome and eosinophilic gastroenteritis remains unknown; therefore, additional case reports of Noonan syndrome with eosinophilic gastroenteritis are required to elucidate this potential relationship.

## Key Message

Noonan syndrome, a genetic disease with a number of symptoms, has not been reported complicated with eosinophilic gastroenteritis. We report the first case eosinophilic gastroenteritis in a female patient with Noonan syndrome with a missense variation in the *PTPN11* gene.

## Background

Noonan syndrome (NS) is a genetic disease with a prevalence of 1 in 1000–2500 live births [1]. It is caused by several genes, with *PTPN11* gene missense variations accounting for nearly 50% of cases [2]. NS may present with a number of symptoms, including a short stature, congenital heart disease, and characteristic facial features because of dysregulation of RAS/MAPK pathway [3].

Eosinophilic gastroenteritis (EGE) is a rare disease that is characterized by eosinophil infiltration of the gastrointestinal tract due to the release of cytokines, such as interleukin-5 (IL-5), which is associated with eosinophil differentiation and proliferation. EGE exhibits various symptoms, including abdominal pain, nausea, vomiting, diarrhea, and weight loss. Regarding affected ages and sex, EGE is found most frequent in middle age (30–50 years), and the sex ratio is reported to be roughly equal. Multiple therapeutic regimens have been used, such as corticosteroids, leukotriene antagonist therapy, and dietary restriction for EGE [4]. 

Although gastrointestinal symptoms occur in NS, there have been no case reports of NS complicated by EGE. We herein describe the first known case of EGE in a female patient with NS with a missense variation in the *PTPN11* gene, including the genetic background; it may suggest a novel genotype–phenotype association.

## Case presentation

The patient was a 14-year-old Japanese girl of non-consanguineous parents who was born at 36 weeks and 4 days of gestation with a birth weight of 3444 g and birth height of 50.0 cm. She was admitted to the neonatal intensive care unit (NICU) in our hospital due to neonatal hypercapnia. Pulmonary artery valve stenosis and an atrial septal defect were detected. She had no allergic diseases. There was no family history of allergic diseases. She had two half-siblings with no underlying diseases.

She was clinically diagnosed with NS on the basis of characteristic facial features, short stature, chest anomalies, developmental delay, and congenital heart diseases at 1 year old. A genetic analysis conducted at the Kazusa DNA Research Institute involved the analysis of the protein-coding regions of the *PTPN11*, *SOS1*, *RAF1*, *RIT1*, *KRAS*, *NRAS*, *SHOC2*, *CBL*, *BRAF*, *SOS2*, *MRAS*, *RRAS*, *LZTR1*, and *RRAS2* genes. The analysis was performed using next-generation sequencing, focusing on rare variation with an allele frequency of 0.1% or lower. The genetic test revealed a missense variation, NM_002834.5:*c.215C > G*/NP_002825.3:*p.Ala72Gly* in the *PTPN11* gene (ClinVar Variation ID: 13,325), which is classified as pathogenic/likely pathogenic on the basis of the ClinVar database and evaluated in accordance with the American College of Medical Genetics and Genomics (ACMG) guidelines. The classification is supported by evidence such as PS1 (same amino acid change with a different nucleotide substitution), PM1 (location in a mutational hotspot region associated with Noonan syndrome), and PP3 (deleterious predictions by multiple *in silico* tools). This finding aligns with the patient’s clinical features, supporting the diagnosis and enhancing our understanding of the genetic basis of the condition.

**Fig. 1:**
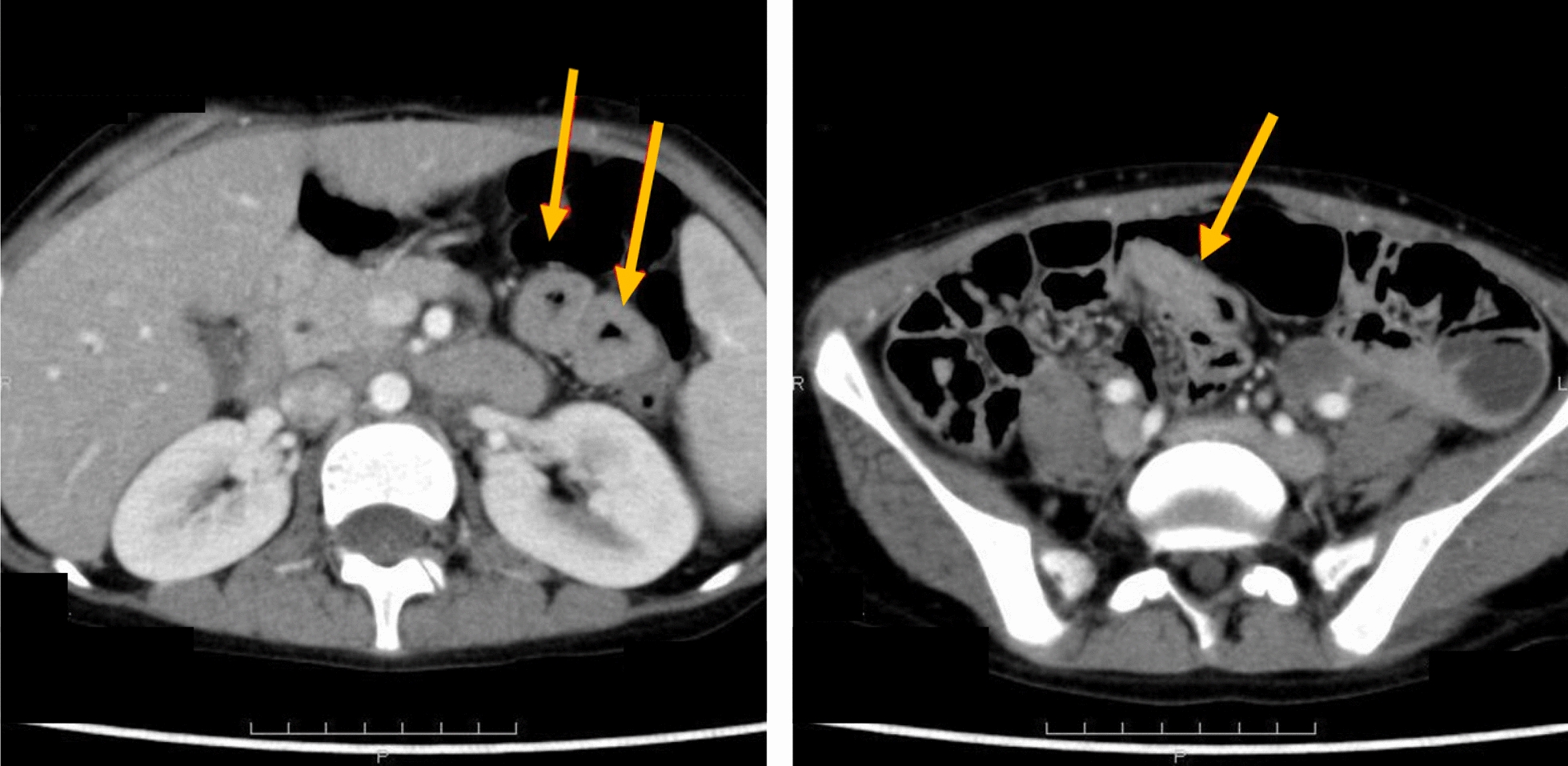
Contrast-enhanced computed tomography of the abdomen: Intestinal wall thickening and lumen narrowing with contrast enhancement, observed from the duodenum to the jejunum, are indicated with arrows

She struggled to adapt to school life from 10 years old onward, and continuously and simultaneously had mild abdominal pain, which was attributed to infectious enteritis, constipation, and stress associated with developmental delay. The patient was hospitalized for abdominal pain and vomiting for 1 week at 12 years old. Blood tests showed a white blood cell count (WBC) of 4700/µL (neutrophils 53.1%, lymphocytes 34.5%, and eosinophils 6.0%) and C-reactive protein (CRP) at 0.02 mg/dL. The patient is not taking any regular medications that may potentially induce eosinophilia in relation to their developmental delays. Contrast-enhanced computed tomography (CT) of the abdomen showed thickening of the intestinal wall and narrowing of the lumen with a contrast enhancement effect from the duodenum to the jejunum, but no neoplastic lesions in the abdominal cavity (Fig. 1).

**Fig. 2:**
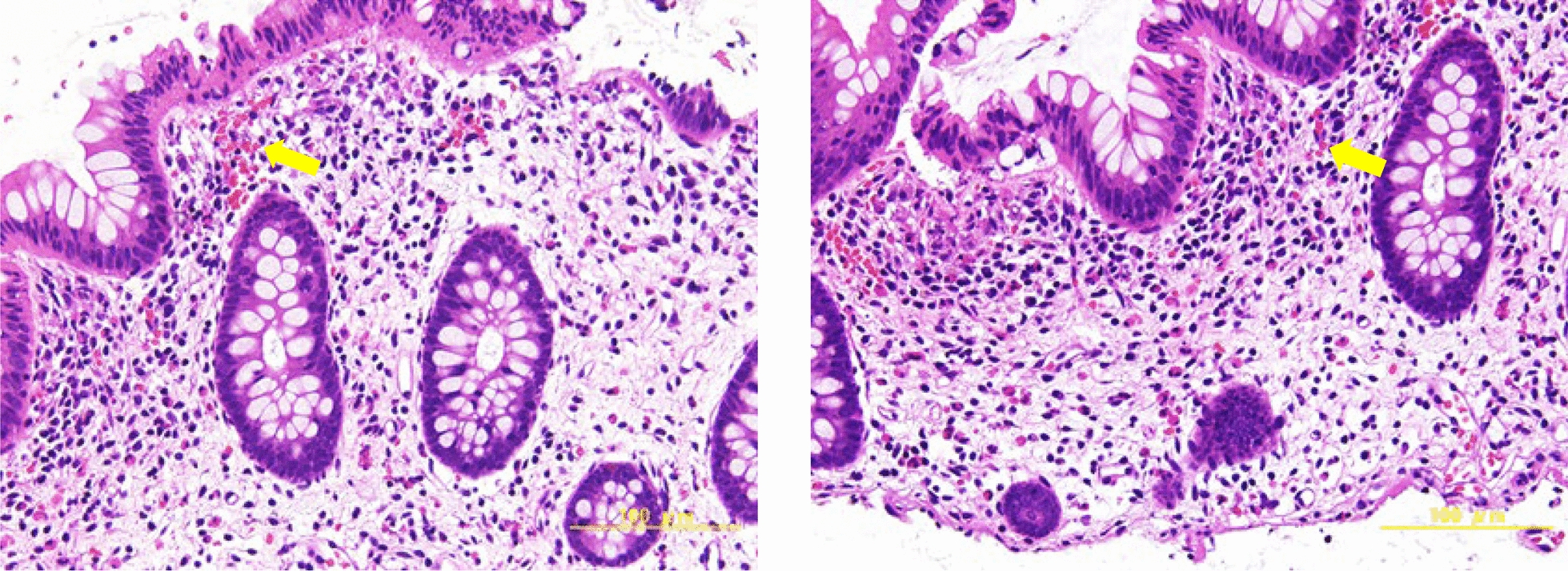
Pathological findings of lower gastrointestinal endoscopy: Multiple foci with an eosinophil count > 20 per high-powered field were detected in the mucosal intrinsic layer of the colon. Eosinophils are abundant in the specimen, and representative ones are indicated with arrows

Abdominal pain and vomiting were relieved by symptomatic medication. The patient was hospitalized due to the recurrence of frequent vomiting and severe abdominal pain 2 weeks after being discharged. Upper and lower endoscopies were performed, and a pathological examination showed multiple foci of eosinophils with an eosinophil count > 20/high power field (HPF) in the mucosal lamina propria of the colon (Fig. 2). EGE was diagnosed, and antihistamines were administered. She underwent second-line investigations, and infections, such as parasitic infections; allergies; drug-induced symptoms/conditions; inflammatory bowel disease; and systemic diseases were considered negative.

She was hospitalized due to vomiting and severe abdominal pain 3 months later. Intravenous prednisolone and leukotriene antagonist therapy attenuated abdominal pain. Vomiting and abdominal pain are currently being controlled by antihistamines and leukotriene antagonist therapy. She underwent a follow-up colonoscopy 2 years later, which revealed chronic inflammation from the jejunum to the rectum and eosinophilic infiltration ranging from 35 to 70/HPF from the jejunum to the transverse colon.

## Discussion

This case of NS was in a 14-year-old girl with recurrent vomiting and abdominal pain. EGE was diagnosed by upper endoscopy and a pathological examination. Abdominal symptoms are being controlled by antihistamines and leukotriene antagonist therapy [5].

EGE is difficult to diagnose because of its nonspecific gastrointestinal symptoms [5]. It is defined by symptoms of abdominal pain and/or diarrhea and the pathological finding of a dense eosinophilic infiltrate in the gastrointestinal wall. EGE is classified on the basis of the site of inflammation, and the threshold for eosinophil count determined by histological findings varies by location. An eosinophil count of ≥ 50/HPF in the colon is one of the diagnostic criteria, and this case was considered eosinophilic colitis on the basis of the clinical symptoms and histological findings [6]. Invasive examinations, such as laparoscopy and/or laparotomy, are often performed because a definitive diagnosis of EGE is difficult due to its nonspecific symptoms [7]. EGE is considered to be related to atopic and allergic factors, and causality appears to be driven by genetic factors, the gut microbiota, and environmental factors, particularly food allergens [8]; however, its etiology remains unknown [9]. Genetic factors related to EGE, such as *TXN*, *PRDX2*, *NR3C1*, *GRB2*, *PIK3C3*, *AP2B1*, and *REPS1*, have been reported [10]. *PTPN11* gene variations may be one of the genetic factors for EGE; however, this needs to be confirmed on the basis of analyses of more NS cases with EGE. There may be an association, which needs further studies to confirm.

The *PTPN11* gene is located on chromosome 12q24.13 in humans [11]. This gene spans approximately 163 kb and consists of 16 exons [12]. The *PTPN11* gene encodes the protein SHP-2 [11], which belongs to the protein tyrosine phosphatase (PTP) family and plays a critical role in intracellular signal transduction [13]. The *PTPN11* gene is the first identified and major NS disease gene, encoding SHP-2, that is found mutated in 50–60% of patients with NS [14]. The *PTPN11* gene variation identified in Noonan syndrome are known as gain-of-function mutations of the RAS/MAPK pathway [15]. The activation of the RAS/MAPK pathway is essential for the differentiation of type-2 helper T cells, which subsequently promotes the production of IL-5 [16]. IL-5 is a key cytokine involved in eosinophilic inflammation and allergic responses, and the development of eosinophilic airway inflammation in a mouse bronchial asthma model are attenuated in transgenic mice by the overexpression of enzymatically inactive Ras molecules in T cells [17]. Therefore, the activation of RAS/MAPK pathway induces the production of IL-5 and allergic disease, such as bronchial asthma. It is well documented that patients with eosinophilic gastroenteritis demonstrate a high prevalence of allergy-related medical conditions, and IL-5 plays a crucial role in the pathogenesis of EGE [18]. Bronchial asthma has been reported as the most prevalent allergic comorbidity, representing more than 50% of cases with allergic history in EGE [19]. These findings suggest a potential pathophysiological link between NS, which results from RAS/MAPK pathway activation due to *PTPN11* pathogenic variation, and eosinophilic disorders.

The codon *Ala72* in the *PTPN11* gene appears to be a mutational hotspot with multiple reported variants, including *p.Ala72Gly*, *p.Ala72Asp*, *p.Ala72Val*, *p.Ala72Ser*, *p.Ala72Thr*, and *p.Ala72Pro* [20, 21]. Functional studies have demonstrated that *p.Ala72Val* and *p.Ala72Ser* promote SHP-2 gain-of-function by destabilizing the catalytically inactive conformation of the protein, leading to prolonged signal flux through the RAS/MAPK pathway [20, 21]. For example, *p.Ala72Val* has been linked to hypertrophic cardiomyopathy and distinctive facial features [20]. In our study, we observed NS with *p.Ala72Gly* variation in the *PTPN11* gene presented with EGE. Further studies, including functional and larger cohort analyses, are needed to confirm these findings and to better understand the full spectrum of clinical manifestations linked to *Ala72* variants.

Although the present case had no allergic history and blood tests did not show an elevated eosinophil count, endoscopic pathology revealed an eosinophilic infiltrate. Difficulties are associated with identifying EGE on the basis of a history of allergy or blood test findings; therefore, we need to consider a thorough examination, including imaging and endoscopy, for recurring abdominal symptoms of NS.

Information on abdominal and allergic symptoms related to NS is limited, and the genetic relationship between EGE and NS currently remains unclear. Although abdominal symptoms in the present case may have been an accidental complication, this is the first case report of NS complicated with EGE. However, the association between NS with *PTPN11* variant and EGE remains purely hypothetical, and the interpretation of causality represents a limitation of this case report.

## Conclusion

We encountered a case of NS with a *PTPN11* gene variant complicated by EGE. A molecular genetic link between *PTPN11* mutations and EGE is suggested; however, the underlying mechanisms remain poorly understood. Further analysis of additional cases will be essential to elucidate this association.

## Data Availability

The data are available from the corresponding author upon reasonable request.
